# Lessons learned during the establishment of a functional National Public Health Institute in the Democratic Republic of Congo: from 2018 to 2024

**DOI:** 10.11604/pamj.2024.49.80.45515

**Published:** 2024-11-18

**Authors:** Karl Angendu, Allan Komakech, José Nyamusore, Sylla Thiam, Ashleigh Howard, Virgile Kikaya, Diene Kaba, Richard Luce, Dieudonné Mwamba, Pierre Akilimali

**Affiliations:** 1 The Democratic Republic of Congo National Public Health Institute, Kinshasa, Democratic Republic of Congo,; 2 Inserm U1094, IRD UMR270, *Université de Limoges, CHU Limoges*, EpiMaCT - Epidemiology of Chronic Diseases in Tropical Zone, Institute of Epidemiology and Tropical Neurology, Omega Health, Limoges, France,; 3 Faculty of Medicine, Christian University of Kinshasa, Kinshasa, Democratic Republic of Congo,; 4 Uganda National Public Health Institute, Kampala, Uganda,; 5 United States Centres for Disease Control and Prevention, Kinshasa, Democratic Republic of Congo,; 6 Johns Hopkins Program for International Education in Gynaecology and Obstetrics, Kinshasa, Democratic Republic of Congo,; 7 Department of Nutrition, Kinshasa School of Public Health, University of Kinshasa, Kinshasa, Democratic Republic of Congo,; 8 Patrick Kayembe Research Center, Kinshasa School of Public Health, University of Kinshasa, Kinshasa, Democratic Republic of Congo

**Keywords:** Health security, National Public Health Institute, public health functions, universal health coverage, Democratic Republic of Congo

## Abstract

The Democratic Republic of Congo (DRC) experiences several disease outbreaks every year. In 2023 alone, the DRC faced outbreaks of Mpox, measles, yellow fever, vaccine-derived polio, malaria, and cholera, alongside humanitarian crises in some regions. Despite the expertise and experience in responding to such epidemics, the timely detection and response to public health emergencies remained a significant challenge, primarily due to challenges in coordination. Following the country´s 10^th^ Ebola viral outbreak from 2018-2020 which led to more than 2,000 deaths, the DRC government committed to establishing a National Public Health Institute (NPHI) to centralize and provide leadership for the public health functions involved in the prevention, detection, and response to disease outbreaks. The NPHI was legally established in April 2022 and began its operations in September 2022 after the appointment of its leadership team. Since then, the country has achieved improved coordination of outbreak response through the establishment of an incident management system and an emergency operations centre, the launch of a coordinated approach to public health research, and enhanced mortality surveillance. Enabling factors for the establishment of the NPHI included political will and strong partnerships with stakeholders. However, challenges during the setup and early phases of its operations, such as resistance to change, delays in mobilising funding, and coordinating support, were also noted. In this paper, we document some of the key lessons learned during the establishment of the NPHI in the DRC, the early successes, how the challenges encountered were addressed, and insights for countries intending to establish their own NPHIs.

## Essay

The Democratic Republic of Congo (DRC) frequently faces public health emergencies, including outbreaks of Ebola virus disease, yellow fever, measles, Mpox, and COVID-19, as well as natural disasters such as floods and volcanic eruptions, often exacerbated by insecurity [1-3]. Before 2022, the country had strategies and mechanisms in place to prevent and manage public health events. However, many public health functions, including surveillance and emergency response, were fragmented across various vertical programs and management structures. This fragmentation led to weak coordination and accountability, hindering the timely detection of and response to public health threats. In an era of increased human mobility, trade, and urbanisation, with complex human-animal-environment interactions, more dynamic and innovative approaches to disease control are required. These efforts aim to establish resilient and sustainable health systems in alignment with the International Health Regulations (IHR) of 2005 [4].

The International Association of National Public Health Institute (IANPHI) and the Africa Centres for Disease Control and Prevention (Africa-CDC), with support from the World Health Organization (WHO), have developed frameworks advocating for the establishment of government-led, science-based National Public Health Institutes (NPHIs). The NPHIs are designed to lead and coordinate essential public health functions such as surveillance, emergency preparedness and response, public health research, public health workforce development, partnership building, identification of emerging public health needs, and mobilization of funds to implement national public health plans [5,6]. In performing these functions, NPHIs ensure compliance with the IHR (2005), strengthen the overall health system through data-driven decision-making, and support the WHO´s long-term vision of achieving the triple billion targets: health security, universal health coverage (UHC), and health promotion [7-9].

**Figure 1:**
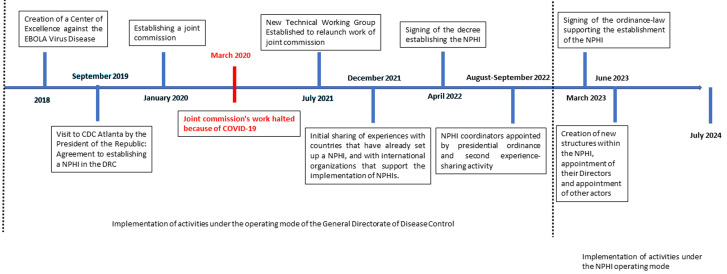
Democratic Republic of Congo-National Public Health Institute development milestones

In 2018, the United States Centres for Disease Control and Prevention (USA-CDC), in collaboration with other partners, began supporting the DRC government in establishing its NPHI, at the government's request [10]. The process of setting up the NPHI in the DRC involved several key steps (Figure 1). This paper aims to present the successes, enablers, and challenges encountered during the establishment of the DRC NPHI, providing insights and lessons learned for other countries intending to establish their own NPHIs.

**Transition from the General Directorate of Disease Control (GDDC) to NPHI and added value:** the process of establishing the NPHI in the DRC began following a visit to the US CDC headquarters in the United States in September 2019 by His Excellency President Felix Tshisekedi. This visit took place during a major Ebola outbreak that had started in 2018. In response, a joint commission composed of officials from the President's office and the Ministry of Health was established in January 2020 to lead the process, under the coordination of the Secretary General of the Ministry of Health. However, in March 2020, the commission´s work was interrupted by the emergence of the COVID-19 pandemic.

After more than a year of delay due to the pandemic, the Ministry of Health resumed the process in July 2021. The Ministry, through the GDDC, which was the previously existing structure, appointed a technical working group (TWG) that included experts from the Ministry of Health, US CDC, and JHPIEGO to provide technical support for the implementation process. This TWG mapped the main public health functions and identified where they were housed to inform the design of the new implementation and the necessary reform processes. The TWG drafted a roadmap and vision for the NPHI´s establishment, including strategic and operational plans, an organizational framework, and an organizational structure (organogram). Simultaneously, the legal processes began in July 2021 to issue gazette a decree for the creation of the NPHI, a key requirement for international recognition.

On April 9^th^, 2022, the DRC-NPHI was officially established by decree n°22/16 [11], which assigned eight key functions to the NPHI: research; workforce development; health promotion and education; disease surveillance, detection, and monitoring; outbreak investigation and control; health information analysis for policy development; and laboratory science, all in alignment with international frameworks [12]. The legal framework was further reinforced by the issuance of ordinance law n°23/006 on March 3^rd^, 2023 [13]. Under this ordinance, the Director General and members of the board of directors were appointed by presidential order. Subsequently, certain directors were appointed by the decision of the board of directors. The NPHI´s creation involved establishing new structures such as the Public Health Emergency Operations Centre (PHEOC), and integrating existing structures, particularly the GDDC in its entirety, ensuring the continuity of key public health functions.

**Figure 2:**
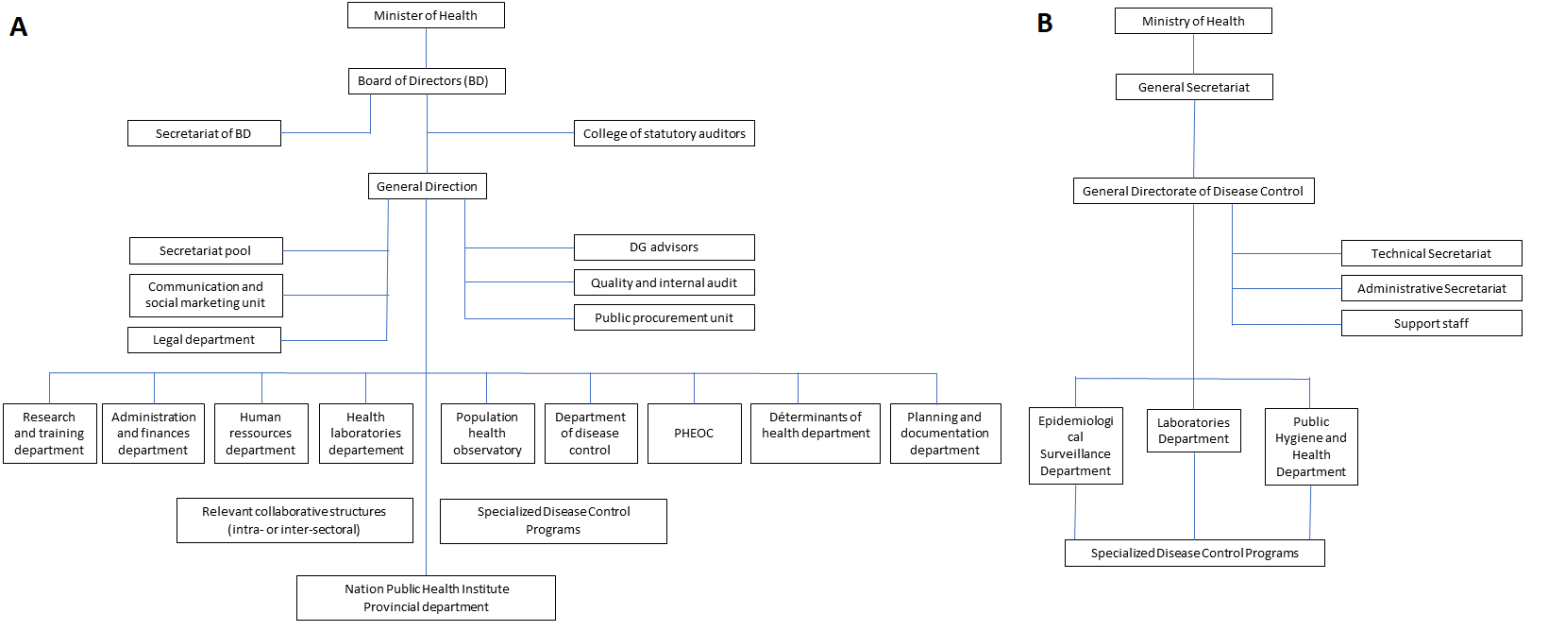
(A, B) comparison of Democratic Republic of Congo-National Public Health Institute and General Directorate Of Disease Control (GDDC) Organogram: Democratic Republic of Congo-National Public Health Institute Organogram

The GDDC and the NPHI in the DRC represent two distinct approaches to public health management. The GDDC was characterised by fragmented programs and structures, with various public health functions dispersed across multiple authorities, leading to limited coordination and accountability. Its primary focus was on disease control without a comprehensive mandate encompassing the broader aspects of public health. In contrast, the NPHI offers a centralised structure with clear roles and responsibilities, significantly enhancing coordination through an incident management system and a PHEOC. The NPHI's broader mandate includes not only disease surveillance and control but also public health research, human resource development, and the implementation of the One Health approach, which integrates human, animal, and environmental health. This centralized and comprehensive framework enables the NPHI to address public health challenges more effectively, ensuring timely detection and response to outbreaks and fostering a more resilient public health system (Figure 2).

**Achievements and benefits of the DRC-NPHI:** the establishment of the NPHI has brought about several significant advancements in the public health landscape of the DRC, which would not have been possible under the previous fragmented system of the GDDC. One of the key achievements of the NPHI is the centralized coordination of research initiatives. While the DRC might have independently initiated research into cardiovascular and metabolic diseases or pilot studies [14] on universal health coverage (UHC) without the NPHI, the institute has provided a cohesive framework that integrates these research efforts across multiple domains. This integration has resulted in more comprehensive and multidisciplinary studies that are better aligned with national health priorities.

In the implementation of the UHC, capacity-building for midwives and other perinatal care providers has been a notable success, facilitated by the coordination provided by the NPHI. The capacity-building efforts within the free maternity and newborn care program benefited from the development of an implementation framework and an innovative conceptual model for training mentees and clinical mentors in UHC. Additionally, research is being conducted to evaluate the effectiveness of this program, an initiative that would have been difficult to realize under the previous GDDC structure [15-17]. Moreover, the NPHI's enhanced coordination capabilities have led to more effective responses to disease outbreaks. The institute's centralized incident management system and PHEOC have enabled quicker and more organized responses to health emergencies. For example, during the Mpox and cholera outbreaks, the NPHI's streamlined surveillance and rapid response teams facilitated timely detection and intervention, reducing the spread and impact of these diseases. Such coordinated and swift responses would have been challenging under the fragmented structure of the previous system.

Additionally, the NPHI has significantly enhanced the integration of public health laboratories and improved the efficiency of sample transport networks. This has resulted in faster confirmation of disease outbreaks and a more robust epidemiological surveillance system. Previously, the GDDC's limited coordination capabilities often led to delays and inefficiencies in laboratory operations and sample handling. The NPHI has also fostered stronger collaborations across various sectors of the health system, thereby improving epidemic preparedness and response. By consolidating public health functions under one umbrella, the NPHI has improved the organization and execution of multisectoral responses to public health emergencies. The successful detection and response to Mpox, cholera, and measles outbreaks are direct outcomes of these improved collaborative efforts, which were less effective under the GDDC's fragmented structure. Given the annual recurrence of these epidemics, the success has been largely due to the systematic introduction of the incident management system by the NPHI as a standardized approach to responding to epidemics and other public health emergencies. This approach has facilitated the mobilization of resources and the effective management of interventions and responders. The efficient organization and management of rapid response teams, managed by the NPHI, have also been key factors in the positive outcome achieved.

**enabling factors of the NPHI establishment:** to strengthen its efforts to respond to and coordinate public health activities, the DRC established its NPHI, joining other NPHIs across the continent that are a part of the IANPHI network [18]. The formation of NPHIs has proven valuable in building collaborative national and international networks essential for their operationalisation [19]. The role of NPHIs has also been well documented, particularly during the COVID-19 pandemic, and is critical for achieving UHC [20].

The relatively swift establishment of the DRC-NPHI was due to several key factors: an inclusive approach, advocacy, and awareness-raising among stakeholders, strong political will to implement systematic change, and commitment at all levels, from the ministerial leadership to the technical experts. Support from the presidency and the Ministry of Health ensured that the necessary legislative and policy frameworks were established. Secondly, engaging legal experts was crucial to preventing legal conflicts with pre-existing vertical programs and ensuring the new NPHI´s independence from inefficient management and decision-making. The frameworks provided by IANPHI and Africa CDC were instrumental in the initial setup of the institute.

Thirdly, early, and continuous stakeholder engagement played a vital role in building consensus and addressing resistance to change. Involving a wide range of stakeholders, including international partners, local health authorities, and community representatives, facilitated a smoother transition and fostered a sense of ownership.

Fourthly, leveraging international frameworks and learning from the experiences of other countries' NPHIs provided valuable insights and guidance. The support and frameworks provided by organisations such as the US CDC, WHO, and African CDC were particularly beneficial. Additionally, strategic partnerships with these organizations and other technical and financial partners were critical for resource mobilization and capacity building. Furthermore, lessons learned from the establishment of other NPHIs, such as the rapid setup of the Liberian NPHI (2 years) and the relatively longer processes to establish the Nigeria CDC (12 years) and Burkina Faso-NPHI, were instrumental [21,22]. Fully operationalizing and sustaining the NPHI requires ongoing engagement and sensitization of all stakeholders throughout its establishment and transition, with a strong emphasis on leadership, governance, and collaboration. Considering the complexity of operationalizing the NPHI and the broader human capital development, as seen in the establishment of other coordinating structures like Senegal's Public Health Emergency Operations Centre (PHEOC) [23], a functional DRC-NPHI necessitates a robust leadership and governance approach. The role of good leadership, financial autonomy, ownership, and political commitment is critical for the success and strengthening of the NPHI [24].

**Challenges during the NPHI establishment in the DRC:** establishing a sustainable NPHI in the DRC is recognized as a complex process that requires flexibility and adaptability to changing circumstances. One major challenge was resistance from key stakeholders, which emerged partly due to insufficient communication and sensitization efforts, leading to misinformation among various actors. To address this, the NPHI implemented extensive communication campaigns and engagement sessions to build awareness and garner support among stakeholders. Another significant challenge was the lack of a clear transition plan for managing human resources, infrastructure, and equipment. The solution involved developing comprehensive transition plans that included capacity-building programs and detailed logistical frameworks to ensure a smooth transfer of responsibilities and resources.

Insufficient government funding posed another hurdle, limiting the NPHI's operational capacity. To overcome this, the NPHI actively pursued innovative funding mechanisms, including public-private partnerships and advocacy for increased budget allocations. Additionally, aligning partner support with NPHI activities proved challenging, as various partners had differing priorities and agendas. The establishment of a coordination mechanism, such as a partner alignment committee, helped streamline efforts and ensure that partner contributions were strategically integrated into the NPHI's operational plans. By addressing these challenges with proactive and strategic solutions, the NPHI strengthened its foundation and improved its ability to fulfill its public health mandate effectively.

Similar challenges were observed during the establishment of other NPHIs, such as those in Uganda and Nigeria [21,25]. Despite these obstacles and considering the dynamic process of building a stronger NPHI, it is essential to recognize that the uniqueness and success of each NPHI reflect the historical, cultural, social, educational, political, and environmental factors of the country. This underscores the importance of country ownership as a crucial element in informing and facilitating health decision-making [26].

**Next steps:** to preserve the achievements made thus far, the NPHI will move to the next phase, focusing on ensuring the full transition and integration of programs, maintaining sustainable technical expertise, building solid partnerships, and achieving financial stability. The DRC government has also designated a site where the future “NPHI complex” will be constructed in the coming years.

Immediate priorities include intensifying public health research activities, establishing a risk communication plan, and developing standard operating procedures for human resource and logistics management. In the medium term, strengthening the public health laboratory system will be critical for the timely detection and confirmation of outbreaks. Furthermore, developing diplomatic strategies to build trust in the NPHI´s sustainability, leadership, and technical and financial accountability, along with establishing partners´ coordination mechanisms, will be crucial to ensure that partners´ efforts are aligned with the NPHI´s operational plan and that investments are strategic and effective. Every five years, the NPHI will conduct a qualitative analysis and policy review of its core functions and attributes, with the first post-NPHI establishment assessment scheduled for 2027.

## Conclusion

In the context of multiple health crises, the widespread prevalence of communicable and non-communicable diseases, and the impact of climate change on health, evident in events like floods and landslides, the creation of an NPHI was crucial for the DRC. The success of a nascent NPHI is based on the implementation of activities that did not previously exist, followed by the integration of those that have been managed by other structures. Significant l achievements have been made in public health research and operations, particularly following the introduction of the PHEOC. The political will behind the NPHI inception paved the path and was maintained throughout the entire process of its establishment. Strong collaborations as well as financial and technical support from partners such as the US CDC, WHO, and Africa CDC ensured a solid foundation of the NPHI. Nonetheless, many challenges were encountered, including stakeholder resistance and funding gaps, which were addressed through improved communication and engagement, as well as the development of a transition plan for human resources.
